# A case control study of environmental and occupational exposures associated with methicillin resistant *Staphylococcus aureus* nasal carriage in patients admitted to a rural tertiary care hospital in a high density swine region

**DOI:** 10.1186/1476-069X-13-54

**Published:** 2014-06-23

**Authors:** Leah Schinasi, Steve Wing, Kerri L Augustino, Keith M Ramsey, Delores L Nobles, David B Richardson, Lance B Price, Maliha Aziz, Pia DM MacDonald, Jill R Stewart

**Affiliations:** 1 Department of Epidemiology, The University of North Carolina at Chapel Hill, Chapel Hill, NC, USA; 2 Department of Infection Control, Vidant Medical Center, Greenville, NC, USA; 3 Division of Infectious Diseases, The Brody School of Medicine at East Carolina University, Greenville, NC, USA; 4 Division of Pathogen Genomics, The Translational Genomics Research Institute, Flagstaff, Arizona, USA; 5 Department of Occupational and Environmental Health, George Washington University, Washington, DC, USA; 6 Social & Scientific Systems, Inc., Durham, NC, USA; 7 Department of Environmental Sciences and Engineering, The University of NC at Chapel Hill, Chapel Hill, NC, USA

**Keywords:** Methicillin resistant *Staphylococcus aureus*, Livestock, Bacterial antibiotic resistance, Concentrated animal feeding operations, North Carolina

## Abstract

**Background:**

Distinct strains of methicillin resistant *Staphylococcus aureus* (MRSA) have been identified on livestock and livestock workers. Industrial food animal production may be an important environmental reservoir for human carriage of these pathogenic bacteria. The objective of this study was to investigate environmental and occupational exposures associated with nasal carriage of MRSA in patients hospitalized at Vidant Medical Center, a tertiary hospital serving a region with intensive livestock production in eastern North Carolina.

**Methods:**

MRSA nasal carriage was identified via nasal swabs collected within 24 hours of hospital admission. MRSA carriers (cases) were gender and age matched to non-carriers (controls). Participants were interviewed about recent environmental and occupational exposures. Home addresses were geocoded and publicly available data were used to estimate the density of swine in residential census block groups of residence. Conditional logistic regression models were used to derive odds ratio (OR) estimates and 95% confidence intervals (CI). Presence of the *scn* gene in MRSA isolates was assessed. In addition, multi locus sequence typing (MLST) of the MRSA isolates was performed, and the Diversilab® system was used to match the isolates to USA pulsed field gel electrophoresis types.

**Results:**

From July - December 2011, 117 cases and 119 controls were enrolled. A higher proportion of controls than cases were current workforce members (41.2% vs. 31.6%) Cases had a higher odds of living in census block groups with medium densities of swine (OR: 4.76, 95% CI: 1.36-16.69) and of reporting the ability to smell odor from a farm with animals when they were home (OR: 1.51, 95% CI: 0.80-2.86). Of 49 culture positive MRSA isolates, all were *scn* positive. Twenty-two isolates belonged to clonal complex 5.

**Conclusions:**

Absence of livestock workers in this study precluded evaluation of occupational exposures. Higher odds of MRSA in medium swine density areas could reflect environmental exposure to swine or poultry.

## Background

Methicillin resistant *Staphylococcus aureus* (MRSA) are resilient and dynamic bacteria. Originally healthcare associated [1], MRSA later emerged in the community, affecting healthy people without recent medical exposures such as hospitalization or surgery [2]. The terms community associated (CA) and healthcare associated (HA) MRSA delineate genetically distinct strains that originally demonstrated equally different epidemiology. The epidemiology of CA and HA MRSA has started to overlap, with CA MRSA causing hospital-onset infections [3] and HA MRSA being carried by people without recent medical exposures [4].

Recently, novel MRSA strains were isolated from livestock and livestock workers [5]. The predominant livestock associated MRSA strains belong to clonal complex (CC) 398 in the Americas and Europe and 9 in Asia [6,7]. Most research on livestock associated MRSA has focused on CC398, which has been identified on livestock and meat products [8] and been shown to be more prevalent in younger rather than older pigs [9,10]. Phylogenetic research suggests that *Staphylococcus aureus* CC398 originated in humans and later spread to livestock [11]. This theory is supported by results showing that a high proportion of livestock associated strains lack the *scn* gene, which is present in nearly all human *S. aureus* isolates and involved in human host immune evasion [12]. Aside from CC398 and 9, other strains, some human associated, are carried by animals [8]. For example, *S. aureus* CC5, a successful human associated strain, was identified in 67% of isolates collected from 49 poultry from across the world [13].

In industrial livestock production, antibiotics are applied subtherapeuticaly, often at the herd level before animals are sick [14]. This creates selective pressures that can foster antibiotic resistance [15]. Thus, the potential for livestock production to serve as a reservoir for human infection is of great interest [11].

Vidant Medical Center (VMC) is an 861-bed teaching hospital affiliated with the Brody School of Medicine at East Carolina University and the tertiary care center for 29 counties in eastern North Carolina, including parts of the densest swine [16] and turkey [17] production areas in the United States. Since February 2007, VMC has screened all patients for MRSA nasal carriage within 24 hours of their admission. MRSA carriers are placed on contact isolation, bathed with chlorhexidine soap, and prescribed the topical antibiotic mupirocin for MRSA eradication [18,19]. The primary objective of this study was to investigate the relationship of nasal MRSA carriage in hospitalized VMC patients with exposures to livestock (pigs, poultry, and cattle), horses, and meat.

## Methods

### Identification of MRSA carriers

Under routine hospital procedures, a double-headed swab (double-headed BBL Culture Swab Liquid Stuart; Becton-Dickinson, Sparks, Maryland) was inserted into the anterior nares of each patients’ nostril and rotated at least 5 times. The swabs were transported to VMC’s Clinical Microbiology Department. One swab was tested for MRSA using the polymerase chain reaction (PCR) with the BD GenOhm® to detect the SCC*mec* gene, which confers methicillin resistance [20]. A positive result from the rapid screen identified patients as MRSA carriers.

### Eligibility criteria and sample size

The following eligibility criteria were applied to cases and controls: age 18-65 years, resident of a top swine producing area in North Carolina, English or Spanish speaker, and screened for MRSA nasal carriage. Cases and controls were restricted to residents of swine producing North Carolina zip codes in which the number of swine permitted for production by the North Carolina Division of Water Quality was equal to or greater than the median for the state. There were 176 eligible zip codes. The age and geographic restrictions were used to increase the prevalence of occupational and livestock exposures.

Sample size goals were based on consideration of participant enrollment time, molecular typing expenses, and estimated numbers of participants needed to achieve 77% - 87% power, assuming 20% - 40% exposure prevalence and an odds ratio of 2.3.

### Case identification

Cases were defined as MRSA nasal carriers, based on a positive result from the rapid PCR screen that was administered at hospital admission. Eligible cases were identified by reviewing daily electronic medical record reports of all admitted patients. The reports listed MRSA screening results, age, gender, and other demographic information.

### Control identification

Controls were non-MRSA carrying patients based on a negative PCR screen. They were identified using the same daily electronic medical records that were used to identify cases. One control was matched to each case based on age (±5 years) and gender. When more than one patient was an eligible match for a case, a random number generator was used to select the potential control.

### Interviews and medical record review

Structured interviews were administered to participants (cases and controls) in their hospital rooms. The questionnaire included information about current employment; job title; work address; number of household members; household member occupation; home address; direct (touching) and indirect (working near but not touching) contact with cows, pigs, chickens, turkeys, and horses at work or outside of work; demographics; ability to smell odor from animal farms when at home; living on a farm with animals; and handling meat at home or at work in the past 2 weeks.

Medical records were reviewed to identify the first-listed diagnosis for the current hospitalization and to determine whether participants were hospitalized for any reason within one year of the current admission.

One author (L.S.) administered all the interviews and abstracted all the data from medical records.

### Geocoding

ArcMap10® (ESRI, Inc., Redlands, CA, USA) was used to geocode home and work addresses. Five participants reported a home address that could not be geocoded; however, the address listed in their medical records was different and could be assigned coordinates. For these 5 participants, coordinates were assigned according to the address in the medical record.

### Human and swine population densities and rural area classifications

Topically integrated geographic encoding and referencing® shapefiles from the 2010 United States Census [21] were used to identify the census block group of each home or work address, and to classify home addresses as being in rural areas; in urban clusters, which contain at least 2,500 people; or in urbanized areas, which contain 50,000 or more people [22]. Urban areas and clusters were combined to form a single urban category.

Densities of total, farrowing, and non-farrowing permitted swine in each census block group (number of swine/square miles in the block group) were calculated using a publicly available database from the North Carolina Division of Water Quality, which lists the type and address of livestock facilities in North Carolina that hold non-discharge wastewater permits, as well the number of permitted animals at each. Farrowing swine include breeding sows and pigs from birth to weaning. Density calculations were categorized by developmental stage because of evidence that livestock associated MRSA is more prevalent in the youngest pigs [9,10].

In addition, 2010 census data was used to assign human population densities to each block group (number of people/square miles in the block group). Block groups are subdivisions of census tracts; in North Carolina, they contain an average of 1,549 residents [21].

Satellite imagery in Google Earth™ was used to identify swine or poultry CAFOs located within 1 mile of each participant’s home and work addresses.

### Bacterial isolates

Duplicate MRSA nasal swabs collected from positive patients were stored at 4°C for up to 48 hours and then transported to VMC’s infection control laboratory. Nasal specimens were streaked onto a CHROMagar® MRSA plate (CHROM agar Microbiology, Paris, France) and incubated for 24 - 48 hours at 37°C. According to manufacturer recommendations, mauve colored colonies were identified as MRSA. One colony from each plate was selected and grown onto sheep’s blood agar (Remel, Lenexa, KS).

### Molecular typing

#### The diversilab® system

Molecular typing of isolates was performed using the Diversilab® system (bioMérieux, Inc., Durham, NC), and conducted according to manufacturer recommendations. DNA was extracted from a pure culture using the UltraClean™ Microbial DNA Isolation Kit (Mo Bio Laboratories, Solana Beach, CA). The NanoDrop® ND-1000 Spectrophotometer (Isogen, Ijssel stein, The Netherlands) was used to estimate the genomic DNA concentration. Sample DNA was diluted to a final concentration of 35 ng/μl.

Repetitive-element based PCR was performed using the Diversilab® *Staphylococcus* kit. Amplicons were separated using a Diversilab® DNA LabChip kit with microfluidic technology, as described previously [18]. The analysis was performed using DiversiLab® software (version v.r.3.3.40). The data for each sample consisted of a dendogram, a virtual gel image (banding pattern), a graph of fluorescence corresponding to each banding pattern, and a similarity matrix. MRSA isolates were classified as CA or HA associated by comparing rep-PCR profiles with samples in the DiversiLab® MRSA library, which contains 70 samples of 14 representative USA pulsed field gel electrophoresis types [23]. Strain relatedness was defined as >95% similarity with up to one band difference in the virtual gel image and determined by the similarity matrix and the pattern overlay function of the DL software. Isolates that did not match any samples in the library according to the above criteria were classified as non-matches.

#### Multi locus sequence typing

Multi locus sequence types (MLST) were assigned using a bash script applied to assembled Illumina data. Briefly, Illumina short read sequences were assembled into contigs using the SPADES assembler [24]. Quality of the assembly was determined by the N50 parameter as well as by mapping that reads back to the assembly [25]. Nucleotide-Nucleotide BLAST (Version 2.2.25+) was used to compare the housekeeping gene against each of the assembled genomes [26]. Sequence similarity matches of genes were determined using thresholds of 100% nucleotide identity and 100% coverage of the query sequence length. The script then used the matched genes and MLST profile data to determine the final MLST type.

#### Eburst

Phyloviz software was used to draw a minimum spanning tree using the *in silico* predicted MLST types of 48 isolates [27]. The plot was drawn to scale.

#### Phylogenetic analysis

MRSA CC398 was not identified among the MRSA isolates collected in this study. However, members of CC5 were identified. A high proportion of broiler chickens have been shown to carry *S. aureus* CC5 [13]. Therefore, Illumina whole-genome sequence data sets were aligned against the chromosome of a published poultry associated sequence type (ST) 5 *S. aureus* reference genome (strain ED98; GenBank accession no. NC_013450 ) using the short-read alignment component of the Burrows-Wheeler Aligner [28]. Each alignment was analyzed for single-nucleotide polymorphisms (SNP) using GATK [29]. To avoid false calls due to sequencing errors, SNP loci were excluded if they did not meet a minimum coverage of 10X and if the variant was present in <90% of the base calls for that position. SNP calls were combined for all of the sequenced genomes such that, for the locus to be included in the final SNP matrix, it had to be present in all of the genomes. SNPs falling in the duplicated regions on the reference genome were discarded.

Phylogenetic trees were generated using the maximum-parsimony method in PAUP v4.0b10 using only the High confidence SNPs. Published CC5 genomes from various sources were used to characterize the nature of the MRSA isolates. Details about the genomes are given in Additional file 1. A published ST80 strain (strain 11819-97; GenBank accession no. NC_017351.1) was selected as an outgroup to root the whole genome sequence tree. Isolates in the clade nearest to this bifurcation point were used to root subsequent trees.

#### scn gene detection

NCBI Blast was used to detect the *scn* gene in the isolate assemblies.

### Statistical analysis

For a number of reasons, not all eligible patients were available to be interviewed- they were discharged from the hospital, unconscious, sleeping, or receiving medical treatments at the time of interviewer contact, for example. Also, not all invited patients agreed to participate. Therefore, after data collection was complete, some participants did not have a matching case or control. To avoid double loss of information in analysis, case and control matched sets were pooled; the gender and age matching case or control who was admitted to the hospital within the shortest amount of time of unmatched participants was selected for the pooled set. Using the same control for more than one case has been described as a valid approach that should not bias measures of association [30,31].

Conditional logistic regression models, which adjusted for the matching variables age and gender, were used to derive odds ratios (OR) and 95% confidence intervals (CI). A term representing education (<high school degree vs. high school degree or more) was entered into all models; this variable was selected *a priori* based on the belief that it could serve as a proxy measure for lifestyle factors that might confound relationships.

Associations between MRSA carriage and the following features and characteristics were explored: residence within 1 mile of a swine or poultry CAFO, swine densities (total, farrowing, and non-farrowing) in the block group of residence, ability to ever smell odor from an animal farm when at home, handling of uncooked meat at work and/or at home in the 2 weeks before hospital admission, indirect contact at work or direct contact at home with horses, indirect contact at work or direct contact at home with livestock (pigs, cows, chickens, turkeys), human population density in the census block group of residence, residence in a rural vs. urban area, living with others vs. alone, and participants’ employment status. Any participant who worked > 0 hours per week within 2 weeks of their hospital admission was considered employed.

Coding decisions were based on variable distributions and comparison of Akaike information criterion statistics. Except variables representing human and swine population density, all exposures were coded as binary terms. Human population density was coded as a linear term. Variables representing densities of total, farrowing, and non-farrowing swine were categorical (0 swine/square-mile, referent vs. > 0 to ≤ 149 swine/square mile vs. > 149 swine/square mile). Zero was the median and mode of the distribution of total swine density and 149 was the 25^th^ percentile of the distribution of observations with non-zero total swine density values.

Because of small numbers within categories of non-farrowing and farrowing swine density, these variables were also categorized according to their own distributions (0 swine/square-mile vs. > 0 to ≤ 77 swine/square-mile vs. > 77 swine/square-mile for farrowing and 0 swine/square-mile vs. > 0 to ≤ 616 swine/square-mile vs. > 616 swine/square-mile for non-farrowing swine). The cut-points 77 and 616 were the median of the distribution of non-zero values for farrowing and non-farrowing densities, respectively.

Models were rerun to compare cases whose nasal swabs grew MRSA colonies with matched controls. Also, isolates were classified as CA or HA based on their USA pulsed field gel electrophoresis (PFGE) types, which were identified by the Diversilab® system. The classification of the USA types as CA or HA was based on the historic origins of these isolates in the United States [23]. CA and HA carriers were compared with matched controls.

Statistical analysis software version 9.3 (Cary, NC) was used to conduct all analyses.

This study was approved by the non-biomedical institutional review board at the University of North Carolina at Chapel Hill (Study #11-0907), and by East Carolina University’s University and Medical Center Institutional Review Board office (Study #11-0257). All participants provided written informed consent and signed Health Information Portability and Accountability Act authorization forms.

## Results

From July - December 2011, 164 cases and 190 controls were invited to participate, and 121 (73.8%) and 122 (64.2%) were enrolled, respectively. Four cases and three controls who reported zip codes outside the eligible areas were subsequently excluded, leaving 117 cases and 119 controls. One hundred (89.3%) matched sets had 1 case and 1 control. After pooling cases and controls to avoid loss of information, 7 (6.3%) matched sets had 2 controls and 1 case and 5 (4.5%) matched sets had 2 cases and 1 control.

### Participant characteristics

Sixty-seven (57.1%) cases and 68 (57.3%) controls were female (Table 1). Twenty-nine (24.4%) controls and 24 (20.5%) cases were 18 – 29 years of age, and 24 (20.5%) cases and 12 (10.9%) controls had less than a high school degree. Cases and controls had similar race/ethnicities; 61 (51.3%) controls and 63 (53.9%) cases were non-white. Ten (8.4%) controls and 2 (1.7%) cases had a first-listed diagnosis for a factor that influenced their health status and contact with professionals—renal donation, medical device placement, etc. Nine (7.6%) controls and 6 (5.1%) cases had a first-listed diagnosis for injury or poisoning; 15 (12.6%) controls and 20 (17.1%) cases had symptoms and ill-defined conditions (chest pain, cough, etc.) listed first.

**Table 1:** **Characteristics of methicillin resistant *Staphylococcus aureus *** **nasal carriers and matched controls hospitalized at Vidant Medical Center in eastern North Carolina, 2011**

	**No. (%)**			
**Characteristics**	**Controls (n** = **119)**		**Cases (n** = **117)**	
Female	68	57.1	67	57.3
Age, years				
18-29	29	24.4	24	20.5
30-39	15	12.6	16	13.7
40-49	17	14.3	22	18.8
50-59	40	33.6	37	31.6
60-65	18	15.1	18	15.4
Non-white race	61	51.3	63	53.9
Less than high school or General Educational Development diploma	13	10.9	24	20.5
Hospitalized within the past year	70	58.8	71	60.7
*First-listed diagnosis for the current hospitalization*				
Injury and poisoning^a^	9	7.6	6	5.1
Factors influencing health status and contact with health professionals^b^	10	8.4	2	1.7
Symptoms, signs, and ill-defined conditions^c^	15	12.6	20	17.1
Live in a block group with any permitted swine	47	39.5	58	49.6
Live on a farm with animals	4	3.4	5	4.3

^a^First-listed diagnosis for the current hospitalization corresponded to International Disease Classification, 9^th^ edition codes 800-999.

^b^First-listed diagnosis for the current hospitalization corresponded to International Disease Classification, 9^th^ edition codesV01-V89.

^c^First-listed diagnosis for the current hospitalization corresponded to International Disease Classification, 9^th^ edition codes 780-799.

Fifty-eight (49.6%) cases and 47 (39.5%) controls lived in a block group with any permitted swine. Five cases (4.3%) and 4 (3.4%) controls lived on a farm where animals were raised. None lived on a farm with confined animals. Participants lived in 152 block groups in eastern North Carolina or the most eastern part of central North Carolina.

### Occupational and environmental exposures

No participants worked directly with livestock. Two (1.7%) cases and 8 (6.7%) controls had a work address within 1 mile of a swine or poultry CAFO. Four (3.4%) cases and 13 (10.9%) controls worked in a census block group with any permitted swine. Four (3.4%) controls and 1 (0.9%) case reported indirect occupational contact with livestock; none were employed at a livestock farm or slaughterhouse. Five (4.2%) controls and 4 (3.4%) cases worked in medical services; 4 (3.4%) controls and 5 (4.3%) cases worked with children.

Four (3.4%) controls and 1 (0.9%) case had a household member who worked on a farm with animals; 2 controls and 1 case reported that animals on the farm where the household member worked lived in confinement. Proportions of controls and cases living with people who worked in healthcare were similar: 15 (12.8%) controls and 12 (10.3%) cases.

The mean ± 1 standard deviation of the densities of total, farrowing, and non-farrowing swine in residential block groups were 400.2 ± 760.7, 44.9 ± 128.6, and 355.3 ± 709.5 swine/square mile, respectively. The mean of the square miles in each of these categories was 6.6 ± 13.2, 28.8 ± 22.8, and 25.0 ± 16.3 swine/square mile, respectively.

Adjusted for education, cases had 4.76 times the odds of living in census block groups with medium densities of swine (>0 - 149 swine/square mile), compared to block groups with no swine (95% CI: 1.36-16.69, Table 2). Associations between case status and residence in block groups with medium densities of farrowing (OR: 1.99, 95% CI: 0.98-4.00) and non-farrowing swine (OR: 2.04; 95% CI: 0.61-6.85) were positive but lower in magnitude. The proportions of cases and controls living in block groups with the highest densities of total and non-farrowing permitted swine were similar. A lower proportion of cases than controls lived in block groups with > 149 farrowing swine per square mile (OR: 0.42; 95% CI: 0.15-1.13). After re-categorizing farrowing and non-farrowing densities according to their own distributions, the lack of a linear dose-response relationship between case status and swine densities remained (data not shown).

**Table 2:** **Estimates of associations of methicillin resistant *Staphylococcus aureus *** **nasal carriage with environmental and occupational exposures among hospitalized patients at Vidant Medical Center in eastern North Carolina, 2011**

	**No. (%)**		**Conditioned on age and gender, adjusted for education**	
	**Controls**	**Cases**	**OR**	**95% CI**
	**(n** = **119)**	**(n** = **117)**		
Permitted swine per square mile of block group				
0	72 (60.5)	59 (50.4)	1.00	-
>0-149	7 (5.9)	20 (17.1)	4.76	1.36-16.69
>149	40 (33.6)	38 (32.5)	0.95	0.53-1.72
Permitted farrowing swine per square mile of block group				
0	87 (73.1)	77 (65.8)	1.00	-
>0-149	17 (14.3)	34 (29.1)	1.99	0.99-4.00
>149	15 (12.6)	6 (5.1)	0.42	0.15-1.13
Permitted non-farrowing swine per square mile of block group				
0	77 (64.7)	70 (59.8)	1.00	-
>0-149	5 (4.2)	10 (8.6)	2.04	0.61-6.85
>149	37 (31.1)	37 (31.6)	0.95	0.54-1.68
Live within 1 mile of a concentrated animal feeding operation				
No	89 (74.8)	94 (80.3)	1.00	-
Yes	30 (25.2)	23 (19.7)	0.60	0.31-1.16
Ever smell odor from a farm with animals when at home				
No	97 (81.5)	86 (73.5)	1.00	-
Yes	22 (18.5)	31 (26.5)	1.51	0.80-2.86
Ever have contact with pigs, chickens, cows, or turkeys^a^				
No	109 (91.6)	112 (95.7)	1.00	-
Yes	10 (8.4)	5 (4.3)	0.52	0.15-1.82
Ever have contact with horses^a^				
No	110 (92.4)	110 (94.0)	1.00	-
Yes	9 (7.6)	7 (6.0)	0.70	0.22-2.21
Ever have contact with uncooked meat products at work or at home				
No	40 (33.6)	44 (37.6)	1.00	-
Yes	79 (66.4)	73 (62.4)	0.81	0.46-1.41
Current member of the work force^b^				
No	70 (58.8)	80 (68.4)	1.00	-
Yes	49 (41.2)	37 (31.6)	0.74	0.41-1.33
Household members present				
No	23 (19.3)	15 (12.8)	1.00	-
Yes	96 (80.7)	102 (87.2)	1.66	0.81-3.39
Live in a rural area^c^				
No	57 (47.9)	54 (46.1)	1.00	
Yes	62 (52.1)	63 (53.9)	0.92	0.55-1.54
Human population density in block group of residence,^d^ mean (std)	1003.2 (1416.0)	920.7 (1346.3)	1.00	0.83-1.21

Abbreviations: odds ratio, OR; confidence interval, CI.

^a^Exposed category includes participants who reported direct contact outside of work and/or indirect contact at work; no participant reported direct contact at work.

^b^Defined as working within the 2 weeks preceding the current hospital admission.

^c^Defined based on address and using 2010 United States Census Bureau definition of rural and urban areas, with urban areas and clusters combined into a single category.

^d^Defined as population/square mile in census block group of residence. Entered into the model as a linear term, and the estimate represents the odds ratio for every increase in 1,000 people/square mile.

A higher proportion of cases than controls reported smelling odor from a farm with animals when they were home (OR: 1.51; 95% CI: 0.80-2.86). Less than half of study participants and a higher proportion of controls than cases were current members of the workforce (0.74; 95% CI: 0.41-1.33). Proportions of cases and controls living in a rural or urban area were similar. Additionally, cases and controls had similar human population densities in their block groups of residence. Cases had lower odds of living within 1 mile of a swine or poultry CAFO (OR: 0.60; 95% CI: 0.31-1.16), of handling raw meat products (OR: 0.74; 95% CI: 0.41-1.33), and of having contact with livestock (OR: 0.52; 95% CI: 0.15-1.82) or horses (0.70; 95% CI: 0.22-2.21).

### Comparison of culture-positive cases with controls

In total, 108 duplicate swabs from the 117 cases were available to be cultured, and 49 (45.4%) grew MRSA colonies on selective media. Conditional logistic models adjusted for education were used to compare culture positive MRSA cases with their 52 matched controls (Additional file 2). The relationship between reported odor from a farm when at home and MRSA carriage remained positive (OR: 2.45; 95% CI: 0.84-7.11). The effect estimates for relationships between medium densities of total swine and farrowing swine and MRSA carriage were positive but imprecise (OR: 4.90; 95% CI: 0.57-42.16 and OR: 2.40, 95% CI: 0.81-7.09). Similar to the full analysis, a lower proportion of cases than controls had indirect contact with livestock at work or direct contact with livestock at home (OR: 0.38, 95% CI: 0.03-4.21). The remaining OR estimates were close to 1 and imprecise.

### Molecular typing

All 49 isolates contained the *scn* gene. Table 3 shows the MLSTs and the PFGE USA types identified using the Diversilab® system. Twenty-two MRSA isolates were ST8. Of these, 19 matched USA300, which is historically a CA strain; 2 of the 22 matched USA500, and 1 did not match any of the USA types. Thirteen of the 49 isolates were ST5; of these, 8 matched USA100, 1 matched USA800, and 4 did not match any of the USA PFGE types in the Diversilab® library. The other MRSA isolates were ST105 (n = 4), ST632 (n = 3), ST36 (n = 1), ST45 (n = 1), ST1 (n = 2), and ST840 (n = 2). The 2 ST840 isolates did not match any of the PFGE USA types. One MRSA isolate was not typeable by MLST but matched USA300.

**Table 3:** Multi locus sequence types and pulsed field gel electrophoresis USA types identified by the Diversilab® system of forty-nine MRSA isolates collected from the anterior nares of hospitalized patients at Vidant Medical Center in eastern North Carolina, 2011

**Multi locus sequence type**	**USA PFGE type**	**No. (%)**
8	300^1^	19 (38.8)
8	500^2^	2 (4.1)
8	No match	1 (2.0)
5	100^2^	8 (16.3)
5	800^2^	1 (2.0)
5	No match	4 (8.2)
105	100^2^	4 (8.2)
632	100^2^	3 (6.1)
36	200^2^	1 (2.0)
45	600^2^	1 (2.0)
1	800^2^	2 (4.1)
840	No match	2 (4.1)
Untypeable	300^1^	1 (2.0)

Abbreviations: MRSA, methicillin resistant *Staphylococcus aureus*, MLST, multi locus sequence type; PFGE, Pulsed field gel electrophoresis.

^1^Historically a community associated strain of MRSA.

^2^Historically a hospital associated strain of MRSA.

**Figure 1:**
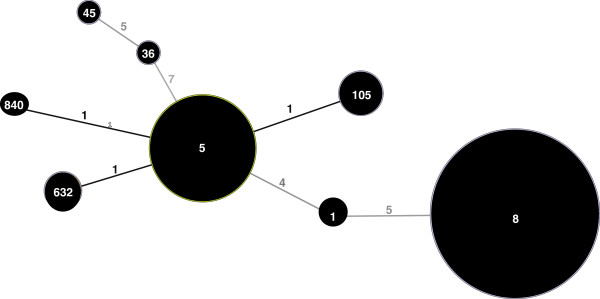
**EBURST plot of 48 methicillin resistant *Staphylococcus. aureus *isolates collected from the anterior nares of hospitalized patients at Vidant Medical Center.** Bubble sizes are proportional to the number of isolates. Numbers inside bubbles represent the multi locus sequence type designations, and numbers on the lines connecting bubbles indicate how many loci were different between one type and another.

An Eburst plot from the profile of the 48 isolates with MLST results indicated that ST840, ST632, and ST105 are single locus variants of CC5 (Figure 1). Twenty-two isolates belonged to this CC. A whole genome sequence-based phylogenetic tree showed that the CC5 isolates from the current study grouped most closely with human isolates from previous studies and distantly from a single chicken isolate. These data do not support poultry as the source of CC5 colonization among the study participants investigated here (Additional file 3).

### Comparison of CA and HA MRSA carriers with controls

CA and HA carriers, classified using the USA PFGE types, were compared to their matched controls using conditional logistic regression models, (Additional file 4). Due to small numbers, the effect estimates were imprecise. CA MRSA carriers had lower odds and HA carriers had higher odds of current employment (OR: 0.44; 95% CI: 0.14-1.44; OR: 1.41, 95% CI: 0.35-5.68) and of living within 1 mile of a CAFO (OR: 0.52, 95% CI: 0.08-3.52; OR: 1.40, 95% CI: 0.23-8.71). CA MRSA carriers had higher odds of reporting odor from a farm when at home (OR: 6.77, 95% CI: 0.80-57.10), and lower odds of living in a rural block group (OR: 0.23, 95% CI: 0.05-1.09) and of handling raw meat (OR: 0.11, 95% CI: 0.01-0.97). HA carriers were similar to controls with respect to these exposures. Compared to controls, HA carriers had higher odds of living in areas with medium densities of total (OR: 2.89, 95% CI: 0.30-28.26) and farrowing swine (OR: 2.45, 95% CI: 0.43-13.89), but similar odds of living in areas with medium densities of non-farrowing swine (OR: 0.97, 95% CI: 0.13-7.44). HA carriers had similar odds of living in areas with high densities of total, farrowing, and non-farrowing swine. Only 1 CA carrier and 0 of their matching controls lived in areas with medium densities of total farrowing swine. No CA carriers or controls lived in areas with medium densities of non-farrowing swine. CA carriers and controls had similar odds of living in areas with medium densities of farrowing swine. CA carriers had lower odds of living in block groups with high densities of total (OR: 0.30, 95% CI: 0.06-1.52), farrowing (OR: 0.41, 95% CI: 0.04-4.55) and non-farrowing swine (OR: 0.40, 95% CI: 0.09-1.71). Due to sparse numbers, the effects of having contact with horses or livestock were not estimable.

## Discussion

MRSA nasal carriage identified within 24 hours of hospital admission by a rapid PCR screen was positively associated with living in areas with moderate densities of swine. A linear dose-response relationship between case status and swine density was absent; proportions of MRSA nasal carriers and controls living in census blocks with the highest swine densities were similar. Furthermore, molecular typing results suggested that participants were not colonized by livestock associated MRSA strains. These results could be explained by unadjusted confounding and/or by the fact that the majority of study participants did not have recent direct contact with livestock.

The overall prevalence of employment was low. Despite restricting the study population to adults ages 18 - 65, less than half were members of the workforce. Higher percentages of controls than cases were employed and hospitalized for injury or poisonings or for factors influencing health status and contact with professionals, rather than for chronic conditions that would indicate poor underlying health. These results are suggestive of a healthy worker effect. To our knowledge, a healthy worker effect in a study of MRSA nasal carriage has not been reported previously. However, in Germany, a higher proportion of hospitalized patients carrying MRSA CC398 versus other strains of MRSA were younger, male, and had shorter lengths of hospital stay [32]. Negative relationships between MRSA carriage and other variables that were considered—contact with livestock or horses and meat handling, for example—might also reflect controls being healthier than cases, since people with poor underlying health would be less inclined or able to engage in such activities.

In previously published analyses of the data from this study, characteristics that could be associated with lower socioeconomic status were positively associated with MRSA carriage--educational status (less than high school or general educational developmental degree versus higher levels) and race/ethnicity (Hispanic and/or non-white race or ethnicity versus white) [19]. Similarly, other studies have reported positive associations between lower socioeconomic status and MRSA [33,34]. A recent population-based study in Pennsylvania found that CA MRSA infection was associated with community economic deprivation [33]. A study in New York City showed that, compared to neighborhoods with a low prevalence of USA300 *S. aureus* infections, neighborhoods with a high prevalence had lower average household income, a higher proportion of residents who were receiving public assistance and were Medicaid eligible, and a higher proportion who were Black or Hispanic [35]. In a study of patients in Baltimore and Atlanta, 51% of patients with CA MRSA infections lived in crowded housing and 65% had a household income of $50,000 or less. Among patients from Atlanta, being Black was associated with CA MRSA infection [36]. In the current study, higher percentages of participants living in block groups with medium densities of livestock were non-white, had less than a high school degree or GED, and were unemployed (data not shown). We did not collect information on household or personal income; however, we adjusted all of the models for educational level, since this variable could be an indicator for socioeconomic status and, in our data, was more strongly predictive of case status than race/ethnicity and employment. Because the variables were highly correlated, we did not include race and employment in the models with education. Nevertheless, adjustment for educational level does not preclude the potential for unadjusted confounding by other variables that might be better indicators of socioeconomic status.

Results from previous studies of the relationship between MRSA carriage and residence near livestock have varied. In Pennsylvania, CA and HA MRSA infections were positively associated with high-density swine production, defined based on distance of the home from the operations and swine count at the facilities [37]. In the Netherlands, 13 of 49 adults living in high pig density areas with livestock contact were MRSA carriers; however, only 1 of 534 (0.2%) people without livestock contact was a MRSA nasal carrier. All MRSA nasal carriers were colonized by the predominant livestock associated strain in Europe, CC398 [38]. In Germany, 0 of 422 students ages 10-16 years not living on pig farms were MRSA carriers [39]. In the Netherlands, a higher proportion of LA MRSA carriers versus carriers of other MRSA strains lived in rural areas, had contact with swine or cattle [40], and lived in municipalities with high densities of swine, cattle, or veal calves [41].

Whereas many studies of MRSA carriage in the United States have been conducted in urban settings [42,43], over 50% of the participants in our study population lived in rural areas. MRSA carriage was not associated with living in rural areas or with human population density in participants’ residential block group. A population-based study in Pennsylvania reported a higher odds of MRSA infection among residents of cities or small towns compared to rural areas [33]. In the Netherlands, Van Loo *et al*. reported a higher prevalence of human associated MRSA strains in areas with high human population densities [40]. However, none of the residential areas in our study were as densely populated by humans as the mostly densely populated areas in the Netherlands. Also, results from a study of MRSA carriage in the United States are not necessarily comparable to one in the Netherlands, where MRSA control measures are more stringent at a national level [44].

In North America and Europe, most research on LA MRSA has reported on CC398 [7,8] although human clones have also been identified on livestock [45]. Of 49 MRSA isolates collected from case participants, none were members of CC398. Recently, *S. aureus* CC398 was found in the nares of 13 of 80 industrial livestock workers in eastern North Carolina [46]. Two of these were MRSA and all were resistant to tetracycline, an antibiotic that has been used for growth promotion in swine since the 1950s [47,48]. Other STs of *S. aureus* that have been found among workers at industrial livestock facilities in eastern North Carolina are ST97, ST20, ST5, ST2546, ST2551, ST188, ST2551, ST15, ST30, ST45, ST1776, ST9, and ST2552 [46]. Of these, only ST5 and ST45 were found in the present study.

Seven of the 49 MRSA isolates in our study did not match any isolates in the Diversilab® system library; 5 of these non-matches were members of CC5 (ST5 and ST840), which is human associated strain of MRSA that is also a common colonizer of poultry [13]. However, phylogenetic analyses indicated that the CC5 members found in this study were not of poultry origin.

All of the 49 isolates contained the *scn* gene, which is generally absent in livestock associated *S. aureus.* Absence of the *scn* gene has been described as an indicator for livestock associated MRSA [46]. The loss of human niche specific genes, such as *scn*, has been posited as the reason that livestock associated strains do not colonize humans as easily as they do animal hosts [11,13]. Also, research has shown that carriage of livestock associated MRSA is related to direct contact with the animals [38,39]. Because of this, and because none of the participants of this study had recent direct contact with livestock, it is not surprising that all of the isolates were *scn* positive and that MRSA CC398 [38,39] was not present. Similar to our findings, in a study in Pennsylvania, MRSA CC398 was not detected among patients with MRSA infections; however, MRSA infection cases were positively associated with indicators of environmental swine exposures [37].

This study had a number of limitations. Patients carrying *S. aureus* resistant to tetracycline but not methicillin would not have been identified as cases in our study and could have been included as controls [32]. Results from this study are not generalizable to non-hospitalized members of the eastern North Carolina community. VMC is the largest hospital in eastern North Carolina. Many of the counties it serves are rural and located in areas with high swine densities. VMC is a member of Vidant Health Systems, which has several smaller affiliated, regional hospitals. For example, Vidant Duplin hospital is located in Duplin County, which contains some of the highest densities of swine in the world. Patients from these rural areas with less severe health problems might have visited the regional Vidant hospitals like Duplin hospital, and as a result, been excluded from the VMC hospitalized population and the current study.

It is unlikely that detection bias entered into this study, since all admitted patients were screened for MRSA. However, participants were not blind to their MRSA carriage status. Knowledge of their screening results could have influenced their responses to questions. Additionally, only 49 cases identified by the PCR assay were confirmed to be MRSA by culture, which could mean that some were false positives. The PCR might have misidentified methicillin susceptible *S. aureus* with remnants of SCC*mec* as MRSA [49] or detected non-viable, non-culturable bacteria [50]. Since the hospital only swabbed the anterior nares, patients carrying MRSA at other locations of their bodies would have been classified as controls. Furthermore, since swabs were tested for MRSA but not methicillin susceptible *S. aureus*, there was a lack of information on bacteria that were susceptible to beta-lactam antibiotics and/or resistant to non-beta lactam antibiotics. This is important considering that, in a recent study of CAFO workers in eastern North Carolina, tetracycline resistant and multi-drug resistant *S. aureus* were more common than MRSA [46]. Cases could have been colonized by multiple MRSA strains [51]; however, DNA of only 1 bacterial colony per culture-positive swabs was extracted and typed. The swine density analysis was limited by the use of block groups as spatial units, meaning that participants in low density block groups might have lived near swine in an adjacent block group, and participants in high density block groups could have lived in a part of the block group with low livestock densities. Finally, variables representing swine or poultry CAFOs within 1 mile radii of addresses might have been misclassified.

This study had several strengths. VMC’s universal screening program provided a convenient way of capturing information on asymptomatic MRSA nasal carriage in residents of eastern North Carolina. The use of the rapid PCR screen allowed for rapid identification and enrollment of MRSA nasal carriers and controls [20]. Also, the PCR technology has been shown to have a higher sensitivity compared to selective media [20]. Information from in hospital interviews and geographic mapping was combined with data from medical records to create a rich data set. Geographic coordinates were assigned according to participants’ reports of their current address, rather than billing addresses that were recorded in medical records. One author (L.S.) performed all of the interviews and abstracted all of the medical record information, which created internal data consistency. There were relatively low amounts of missing data and high participation rates.

## Conclusions

In hospitalized patients, moderate densities of swine in the block group of residence were associated with MRSA nasal carriage detected by PCR. This finding is supported by past evidence of associations between MRSA nasal carriage and contact with swine production. However, unadjusted confounding by socioeconomic factors could explain the observed relationship. No participants had direct contact with livestock, and molecular typing analyses suggested that cases were not colonized by livestock associated strains of MRSA. Similar investigations to this, but with in- and out-patients at smaller regional hospitals in eastern North Carolina would be useful. Active surveillance for novel strains of MRSA is essential, especially at VMC, which is the largest hospital in a region with dense populations of CAFOs. This study provides useful information for designing future studies of the spread of antibiotic resistant bacteria from CAFOs into human communities.

## Abbreviations

CA: Community associated; CAFO: Concentrated animal feeding operations; CC: Clonal complex; CI: Confidence interval; HA: Healthcare associated; LA: Livestock associated; MRSA: methicillin resistant *Staphyloccocus aureus*; MLST: Multi locus sequence type; OR: Odds ratio; PCR: Polymerase chain reaction; PFGE: Pulsed field gel electrophoresis; VMC: Vidant medical center; ST: Sequence type.

## Competing interests

Keith Ramsey has provided consultancy for BD GeneOhm, is on the Speakers Bureaus of Cubist Pharmaceuticals and Sanofi-Pasteur, and has served as an expert witness in legal proceedings related to healthcare-associated infections, and received fees for his services. We have no other interests to report.

## Authors’ contributions

LS, SW, KR, DR, JS, and PM designed the study. LS, SW, and KR designed the study materials. DN, KR, and LS designed participant recruitment methods. LS administered the interviews, reviewed the medical records, geocoded addresses, cleaned the data, and performed the statistical analyses. KA performed the typing and analysis of the isolates with the Diversilab® system. MA performed multi locus sequence typing (MLST) of the isolates, assessed MRSA isolates for presence of the *scn* gene, and conducted the phylogenetic analysis. LP oversaw MLST, *scn* gene assessment, and phylogenetic analyses, and helped to interpret results from these. LS, SW, KA, KR, DN, DR, PM, JS, LP, and MA contributed to the preparation of the manuscript. All authors read and approved the final manuscript.

## Supplementary Material

Additional file 1 **Details of the clonal complex 5 genomes used to characterize methicillin resistant *Staphylococcus aureus* isolates.** Description of data: Table describing clonal complex 5 genomes Click here for file

Additional file 2 **Estimates of association of methicillin resistant *Staphylococcus aureus* nasal carriage identified by culture with environmental and occupational exposures among hospitalized patients at Vidant Medical Center in eastern North Carolina, 2011.** Description of data: Table showing odds ratio estimates and 95% confidence intervals of relationships of environmental and occupational exposures with culture positive MRSA carriage. Click here for file

Additional file 3 **Whole genome sequence-based *Staphylococcus aureus *** **clonal complex (CC5) phylogenetic tree.** A maximum parsimony tree was generated from whole genome SNP profiles of *Staphylococcus aureus* isolates from the current study along with those from previous studies to evaluate potential poultry origins for the isolates colonizing hospitalized patients at Vidant Medical Center in 2011. Details of CC5 genomes used to characterize the isolates are given in Additional file 1. Description of data: Figure showing whole genome sequence based phylogenetic tree. Click here for file

Additional file 4 **Estimates of association of community associated and hospital associated methicillin resistant *Staphylococcus aureus *** **(MRSA) nasal carriage with environmental and occupational exposures among hospitalized patients at Vidant Medical Center in eastern North Carolina, 2011.** Description of data: Table showing odds ratio estimates and 95% confidence intervals of relationships of environmental and occupational exposures with community associated or hospital associated MRSA carriage. Click here for file
