# Supplementary material for: A case control study of environmental and occupational exposures associated with methicillin resistant Staphylococcus aureus nasal carriage in patients admitted to a rural tertiary care hospital in a high density swine region
Source: Environ Health. 2014 Jun 23;13:54. doi: 10.1186/1476-069X-13-54 (PMC4083368; doi:10.1186/1476-069X-13-54)
Supplement: Additional file 1 — Details of the clonal complex 5 genomes used to characterize methicillin resistant Staphylococcus aureus isolates. Description of data: Table describing clonal complex 5 genomes [file 1476-069X-13-54-S1.docx]

| **Additional file 1**. Details of the clonal complex 5 genomes used to characterize the methicillin resistant *Staphylococcus aureus* isolates | | | | | | | | |
| --- | --- | --- | --- | --- | --- | --- | --- | --- |
| **Strain** | **Country of origin** | **Year of isolation** | ***Spa* type** | **Sequence type** | **SCCmec** | **Clonal complex** | **Genbank Accesion number** | **Source** |
| N315 | Japan | 1982 | t002 | ST5 | II | CC5 | BA000018 | HA |
| Mu50 | Japan | 1997 | t002 | ST5 | II | CC5 | BA000017 | HA |
| Mu3 | Japan |  | t002 | ST5 | II | CC5 | NC_009782 | HA |
| JH1 | USA | 2000 | t002 | ST105 | II | CC5 | CP000736 |  |
| JH9 | USA | 2000 | t002 | ST105 | II | CC5 | NC_009487 |  |
| ED98 | Nothern Ireland | 1996-1997 | t002 | ST5 | MSSA | CC5 | NC_013450 | LA |
| 04-02981 | Germany | 2004 | t003 | ST225 | II | CC5 | NC-017340 | HA |
| ECT-R 2 | Sweden |  | t002 | ST5 | MSSA (SCCmec remnant) | CC5 | NC_017343 | HA |
| 10388 | Switzerland | 2001 |  | ST228 | I | CC5 | HE579059 | HA |
| Abbreviations: spa, staphylococcal protein a; SCCmec, staphylococcal cassette chromosome mec; t, type; ST, sequence type; CC, clonal complex; HA, hospital associated; LA, livestock associated | | | | | | | | |
