# Supplementary material for: A case control study of environmental and occupational exposures associated with methicillin resistant Staphylococcus aureus nasal carriage in patients admitted to a rural tertiary care hospital in a high density swine region
Source: Environ Health. 2014 Jun 23;13:54. doi: 10.1186/1476-069X-13-54 (PMC4083368; doi:10.1186/1476-069X-13-54)
Supplement: Additional file 3 — Whole genome sequence-based Staphylococcus aureus clonal complex (CC5) phylogenetic tree. A maximum parsimony tree was generated from whole genome SNP profiles of Staphylococcus aureus isolates from the current study along with those from previous studies to evaluate potential poultry origins for the isolates colonizing hospitalized patients at Vidant Medical Center in 2011. Details of CC5 genomes used to characterize the isolates are given in Additional file 1. Description of data: Figure showing whole genome sequence based phylogenetic tree. [file 1476-069X-13-54-S3.doc]

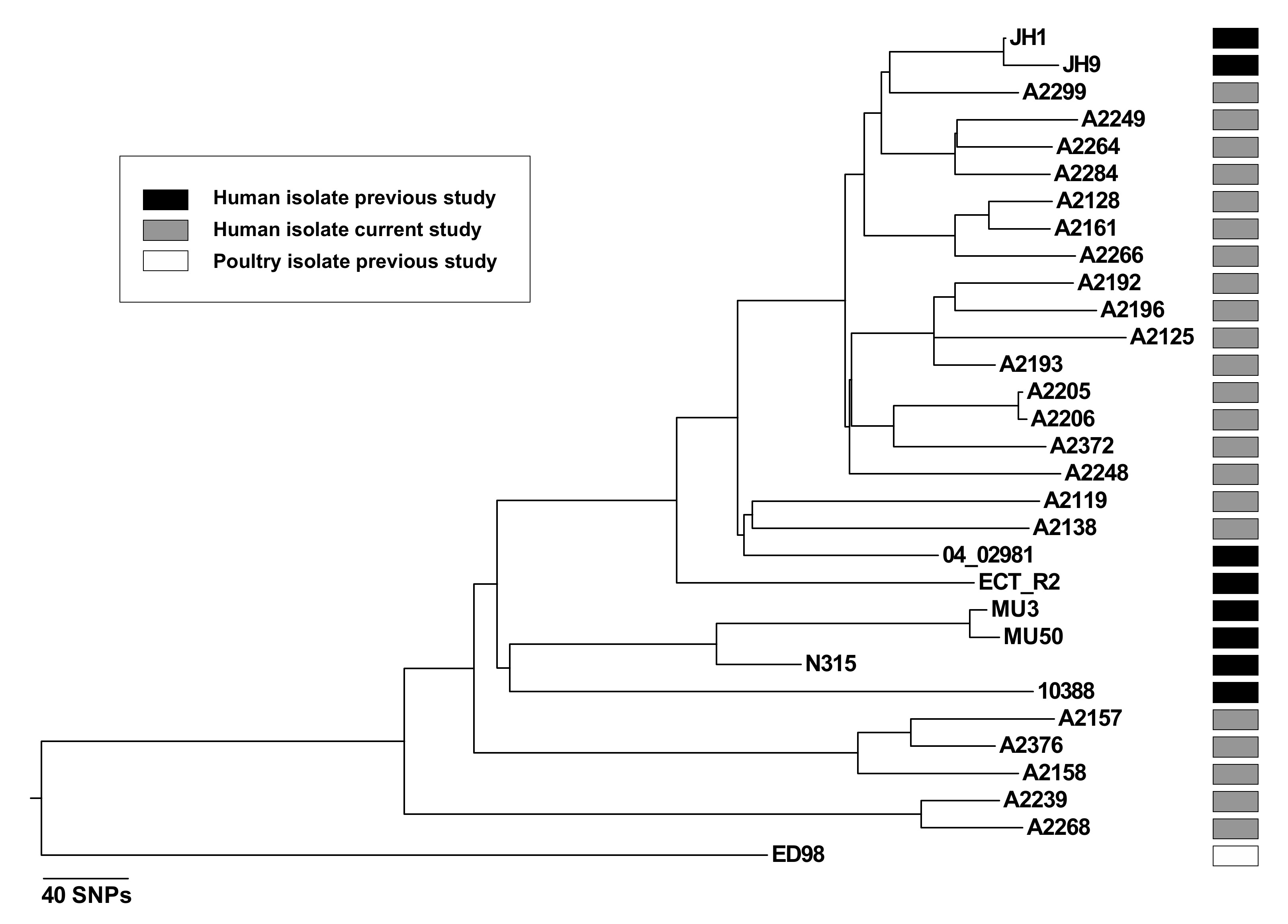


Additional file 3. Whole genome sequence-based *Staphylococcus aureus* clonal complex (CC5) phylogenetic tree. A maximum parsimony tree was generated from whole genome SNP profiles of *Staphylococcus aureus* isolates from the current study along with those from previous studies to evaluate potential poultry origins for the isolates colonizing hospitalized patients at Vidant Medical Center in 2011. Details of CC5 genomes used to characterize the isolates are given in Additional file 1.
