# Supplementary material for: A case control study of environmental and occupational exposures associated with methicillin resistant Staphylococcus aureus nasal carriage in patients admitted to a rural tertiary care hospital in a high density swine region
Source: Environ Health. 2014 Jun 23;13:54. doi: 10.1186/1476-069X-13-54 (PMC4083368; doi:10.1186/1476-069X-13-54)
Supplement: Additional file 4 — Estimates of association of community associated and hospital associated methicillin resistant Staphylococcus aureus (MRSA) nasal carriage with environmental and occupational exposures among hospitalized patients at Vidant Medical Center in eastern North Carolina, 2011. Description of data: Table showing odds ratio estimates and 95% confidence intervals of relationships of environmental and occupational exposures with community associated or hospital associated MRSA carriage. [file 1476-069X-13-54-S4.docx]

| **Additional file 4**. Estimates of association of community associated and hospital associated methicillin resistant *Staphylococcus aureus* (MRSA) nasal carriage with environmental and occupational exposures among hospitalized patients at Vidant Medical Center in eastern North Carolina, 2011 | | | | | | |
| --- | --- | --- | --- | --- | --- | --- |
|  | **Community associated MRSA** | | | **Hospital associated MRSA** | | |
|  | **Controls**  **(n = 23)** | **Cases**  **(n = 20)** | **Conditioned on age and gender, adjusted for education** | **Controls**  **(n = 23)** | **Cases**  **(n = 22)** | **Conditioned on age and gender, adjusted for education** |
|  | **No. (%)** | **No. (%)** | **OR (95% CI)** | **No. (%)** | **No. (%)** | **OR (95% CI)** |
| Permitted swine per square mile of block group |  |  |  |  |  |  |
| 0 | 11 (47.8) | 13 (65.0) | 1.00 | 14 (60.9) | 12 (54.6) | 1.00 |
| >0-149 | 0 | 1 (5.0) | NE | 1 (4.4) | 3 (13.6) | 2.89 (0.30-28.26) |
| >149 | 12 (52.2) | 6 (30.0) | 0.30 (0.06-1.52) | 8 (34.5) | 7 (31.8) | 0.86 (0.27-2.72) |
| Permitted farrowing swine per square mile of block group |  |  |  |  |  |  |
| 0 | 16 (69.6) | 14 (70.0) | 1.00 | 18 (78.3) | 14 (63.6) | 1.00 |
| >0-149 | 3 (13.0) | 5 (25.0) | 1.28 (0.26-6.17) | 4 (17.4) | 7 (31.8) | 2.45 (0.43-13.89) |
| >149 | 4 (17.4) | 1 (5.0) | 0.41 (0.04-4.55) | 1 (4.4) | 1 (4.6) | 0.96 (0.05-18.49) |
| Permitted non-farrowing swine per square mile of block group |  |  |  |  |  |  |
| 0 | 12 (52.2) | 14 (70.0) | 1.00 | 14 (60.9) | 13 (59.1) | 1.00 |
| >0-149 | 0 | 0 | - | 2 (8.7) | 2 (9.1) | 0.97 (0.13-7.44) |
| >149 | 11 (47.8) | 6 (30.0) | 0.40 (0.09-1.71) | 7 (30.4) | 7 (31.8) | 0.94 (0.30-2.90) |
| Live within 1 mile of a concentrated animal feeding operation |  |  |  |  |  |  |
| No | 17 (73.9) | 15 (75.0) | 1.00 | 20 (87.0) | 18 (81.8) | 1.00 |
| Yes | 6 (26.1) | 5 (25.0) | 0.52 (0.08-3.52) | 3 (13.0) | 4 (18.2) | 1.40 (0.23-8.71) |
| Ever smell odor from a farm with animals when at home |  |  |  |  |  |  |
| No | 20 (87.0) | 13 (65.0) | 1.00 | 20 (87.0) | 19 (86.4) | 1.00 |
| Yes | 3 (13.0) | 7 (35.0) | 6.77 (0.80-57.10) | 3 (13.0) | 3 (13.6) | 1.00 (0.20-4.96) |
| Ever have contact with pigs, chickens, cows, or turkeys^a^ |  |  |  |  |  |  |
| No | 21 (91.3) | 20 (100.0) | 1.00 | 22 (95.7) | 20 (90.9) | 1.00 |
| Yes | 2 (8.7) | 0 | NE | 1 (4.4) | 2 (9.1) | NE |
| Ever have contact with horses^a^ |  |  |  |  |  |  |
| No | 21 (91.3) | 20 (100.0) | 1.00 | 23 (100) | 20 (90.9) | 1.00 |
| Yes | 2 (8.7) | 0 | NE | 0 | 2 (9.1) | NE |
| Contact with uncooked meat products at work or at home^a^ |  |  |  |  |  |  |
| No | 5 (21.7) | 10 (50.0) | 1.00 | 7 (30.4) | 6 (27.3) | 1.00 |
| Yes | 18 (78.3) | 10 (50.0) | 0.11 (0.01-0.97) | 15 (68.2) | 16 (76.2) | 1.13 (0.34-3.74) |
| Current member of the work-force^b^ |  |  |  |  |  |  |
| No | 11 (47.8) | 15 (75.0) | 1.00 | 15 (65.2) | 13 (59.1) | 1.00 |
| Yes | 12 (52.2) | 5 (25.0) | 0.44 (0.14-1.44) | 8 (34.8) | 9 (40.9) | 1.41 (0.35-5.68) |
| Household members present |  |  |  |  |  |  |
| No | 4 (17.4) | 3 (15.0) | 1.00 | 4 (17.4) | 3 (13.6) | 1.00 |
| Yes | 19 (82.6) | 17 (85.0) | 1.49 (0.22-9.92) | 19 (82.6) | 19 (86.4) | 1.31 (0.28-6.19) |
| Live in a rural area^c^ |  |  |  |  |  |  |
| No | 7 (30.4) | 12 (60.0) | 1.00 | 13 (56.5) | 11 (50.0) | 1.00 |
| Yes | 16 (69.6) | 8 (40.0) | 0.23 (0.05-1.09) | 10 (45.5) | 11 (50.0) | 1.21 (0.35-4.16) |
| Human population density in block group of residence,^d^ mean (std) | 780.9 (1810.3) | 1325.1 (1746.5) | 1.20 (0.83-1.74) | 1145.1 (1375.2) | 697.5 (944.2) | 0.76 (0.46-1.25) |

Abbreviations: odds ratio, OR; confidence interval, CI; NE, non-estimable

^a^Exposed category includes participants who reported direct contact outside of work and/or indirect contact at work; no participant reported direct contact at work.

^b^Defined as working within the 2 weeks preceding the current hospital admission.

^c^Defined based on address and using 2010 United States Census Bureau definition of rural and urban areas, with urban areas and clusters combined into a single category.

^d^Defined as population/square mile in census block group of residence. Entered into the model as a linear term, and the estimate represents the odds ratio for every increase in 1,000 people/square mile.
